# Case Report: Abiraterone in brain metastases from prostate cancer

**DOI:** 10.3389/fonc.2025.1555897

**Published:** 2025-04-11

**Authors:** Tobias Peres, Stefanie Aeppli, Stefanie Fischer, Christian Rothermundt

**Affiliations:** ^1^ Department of Medical Oncology and Hematology, Cantonal Hospital St. Gallen (KSSG), St. Gallen, Switzerland; ^2^ Department of Medical Oncology and Cancer Centre, Cantonal Hospital Lucerne (LUKS), Lucerne, Switzerland

**Keywords:** prostate cancer, metastatic, brain metastases, cerebral metastasis, hormonesensitive, abiraterone, blood-brain barrier, case report

## Abstract

**Background:**

Prostate carcinoma (PC) is the most common cancer in men worldwide. However, brain metastases (BM) from prostate cancer are extremely rare events, usually in the later course of the disease. There is no established standard of care treatment for this situation. The efficacy of androgen synthesis inhibitor abiraterone in BM from PC is unknown.

**Case:**

We herein report the case of an 83-year-old patient with metastatic hormone-sensitive PC who had multiple BM at primary diagnosis, clinically manifesting with dizziness, ataxia, and unsteady gait. Combination of abiraterone and androgen deprivation therapy showed exceptional sustained cerebral tumor response. After 12 months of treatment, the patient is asymptomatic with an excellent performance status.

**Conclusion:**

Symptomatic BM from PC is a rarity but can show sustained response to abiraterone and androgen deprivation therapy. After comprehensive literature search, there is no comparable case published to date.

## Graphical Abstract

Timeline illustrating response to treatment biochemically and radiographically.

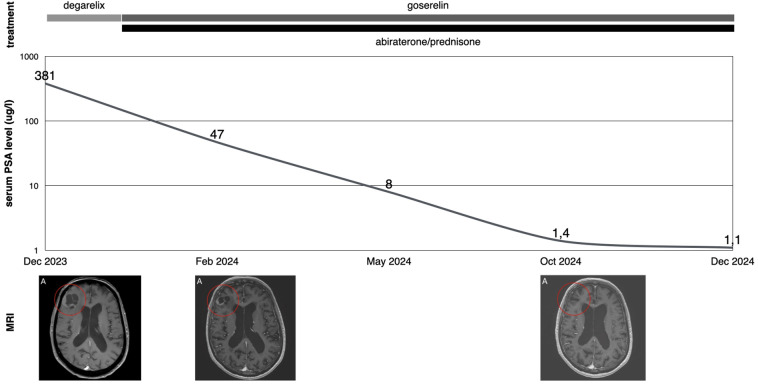

## Introduction

Globally, prostate carcinoma (PC) is the most common cancer in men (1). The majority of patients with metastatic PC present with bone metastases, whereby lymph node and lung metastases are also common (2). However, the occurrence of brain metastases (BM) in metastatic PC is rare (3, 4). BM are much more common in other cancer types, especially lung cancer, breast cancer, and melanoma (5). If BM occur in PC, this is usually in the advanced, pre-treated, later course of disease with castration resistance (6, 7). Of note, a higher incidence of BM has been described in the post-docetaxel compared to the pre-docetaxel treatment era. The higher incidence probably reflects a survival gain due to the introduction of new drugs over the years (8). It is known that docetaxel is virtually unable to cross the blood–brain barrier (BBB), so BM seem more likely to occur if the tumor is otherwise controlled and the patient lives longer (9). Additionally, more sensitive methods of detection, including improved imaging, of BM and other central nervous system (CNS) manifestations, for example, leptomeningeal carcinomatosis, have been routinely used over time in advanced PC (10). Data on exposure to new hormonal agents (NHA) prior to the development of BM are scarce. In a retrospective institutional analysis of 6,596 PC cases with 29 confirmed cases of CNS metastases from PC, 80% of patients had received abiraterone or enzalutamide prior to the development of BM, of whom 50% had ≥6 months of NHA exposure (7). Another retrospective single-center analysis, however, could not show that longer NHA (abiraterone, enzalutamide) exposure duration was associated with more frequent development of visceral metastases and BM after 2011 (11). Histological differentiations, which are distinct from adenocarcinoma, are more likely to be associated with BM. The probability of BM is therefore higher with neuroendocrine dedifferentiated or small cell carcinoma than with adenocarcinoma of the prostate (12). Patients with BM from PC have a poor prognosis. In most cases, survival is no more than a few months (7, 13). Due to the rarity of BM in PC patients, there are no established systemic or intrathecal treatment options. Local treatment strategies (radiotherapy, surgery) for BM are commonly used (14). Pivotal phase 3 trials, which have shown a survival benefit of the androgen biosynthesis inhibitor abiraterone and prednisone over placebo when added to androgen-deprivation therapy (ADT) in the metastatic hormone-sensitive setting, have excluded patients with BM (15). We here present a case with impressive efficacy of treatment combination of abiraterone and ADT in BM from PC with ongoing response after 12 months of treatment. This case is thus unique among published literature.

## Case description

**Figure 1:**
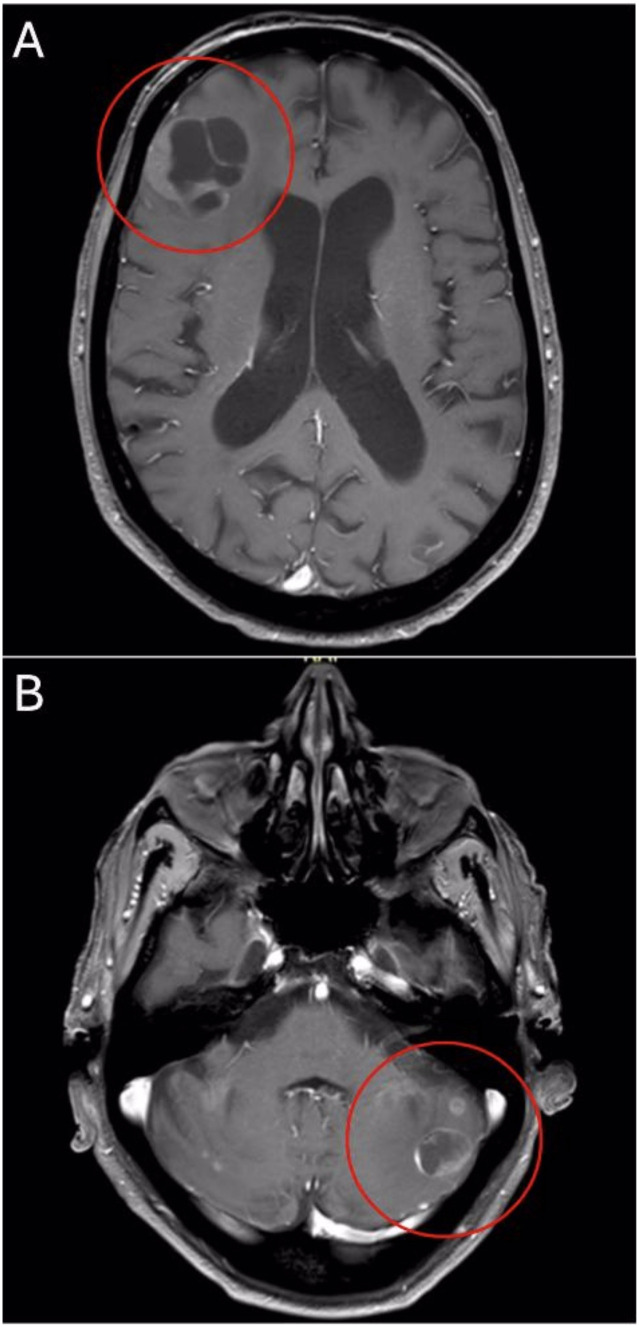
Axial T1-weighted MRI shows right frontal **(A)** and left cerebellar **(B)** lesions at primary diagnosis.

An 83-year-old man presented to our emergency department with dizziness, ataxia, and unsteady gait. The clinical examination revealed truncal and gait ataxia with impaired stance. The patient showed a conspicuous Unterberger test (with rotation to the left side >45°) and a dysmetric finger-nose test with intention tremor. There was a mild diffuse pain on pressure of the entire spine. Due to the neurological symptoms in particular, the patient required hospital admission with constant assistance, corresponding to a Karnofsky Performance Status of 40%. Upon further assessment, a magnetic resonance imaging (MRI) of the CNS showed multiple BM with the two largest lesions located in the right frontal (3.5 cm, **Figure 1A**) and left cerebellar (1.8 cm, **Figure 1B**) regions, each surrounded by significant cerebral edema. In addition, computed tomography (CT) of the thorax, abdomen, and pelvis showed multiple osseous, lymph node, and peritoneal metastases with suspected PC infiltrating the urinary bladder and seminal vesicles. An axillary lymph node on the right was noticeably enlarged, measuring at a short axis of 15 mm. Laboratory tests showed a serum prostate-specific antigen (PSA) level of 381 µg/l (normal range <2.0 µg/l). Serum alkaline phosphatase (ALP) and lactate dehydrogenase (LDH) levels were only slightly elevated at 129 U/l (normal range <120 U/l) and 298 U/l (normal range <265 U/l), respectively. The patient’s medical record revealed no significant pre-existing diseases. Family history was positive with a brother also suffering from PC. The patient had no regular PSA screening.

**Figure 2:**
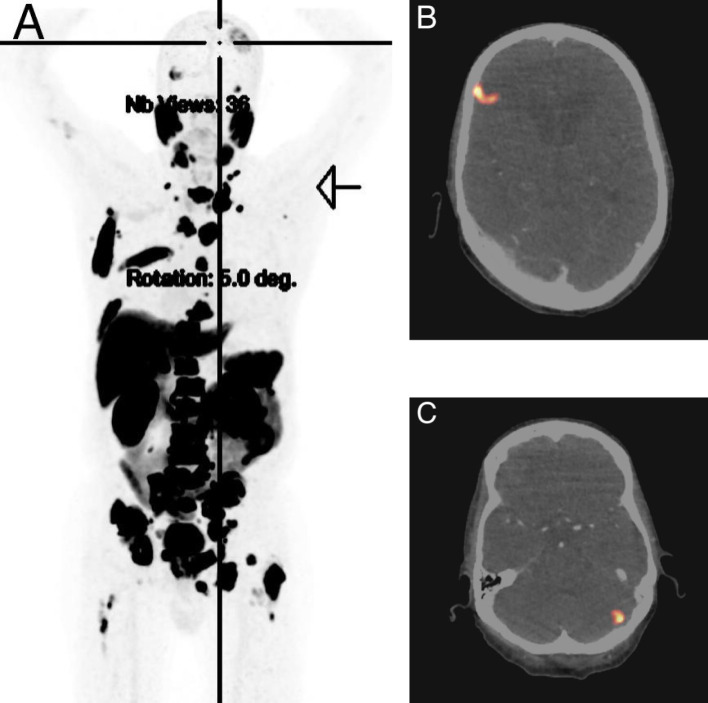
PSMA PET scout image **(A)** and PSMA avid right frontal **(B)** and left cerebellar **(C)** lesions.

**Figure 3:**
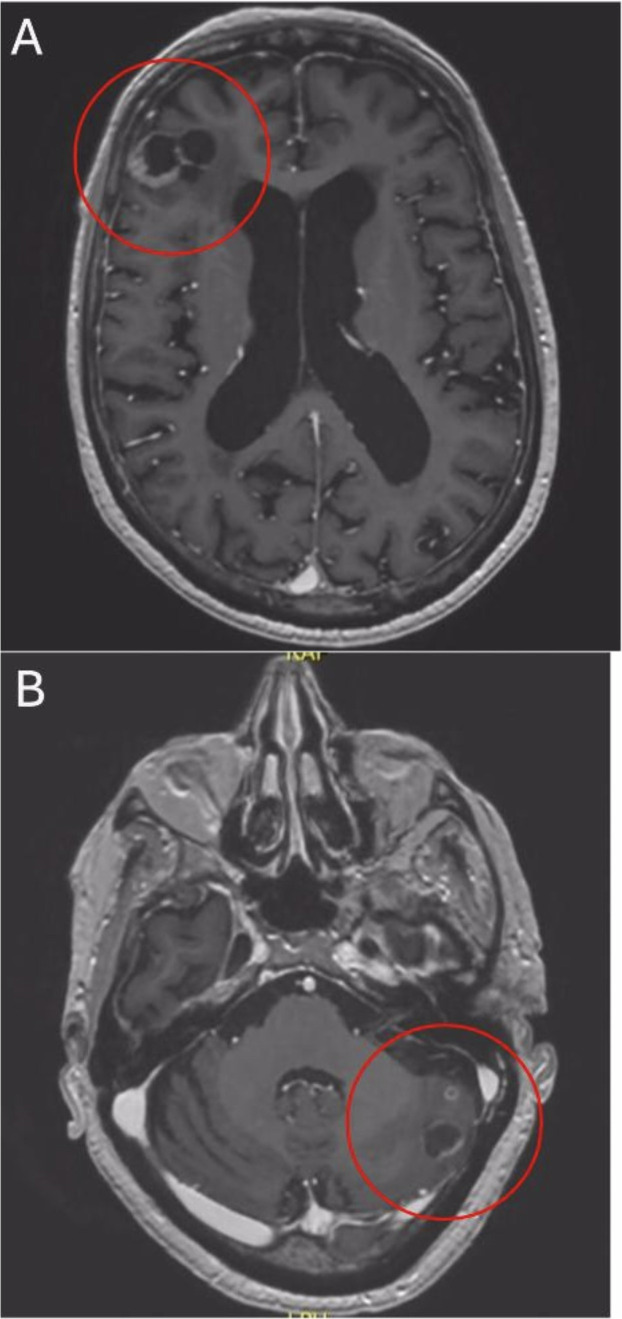
After 5 weeks of treatment axial T1-weighted MRI reveals remission of right frontal **(A)** and left cerebellar **(B)** lesions.

For treatment of perifocal edema, dexamethasone was administered. Initially, due to the massively elevated serum PSA level along with MRI and CT findings including large bone metastases in the spine, GnRH antagonist degarelix (2 × 120 mg) was given to prevent testosterone flare and potential spinal cord compression. According to orthopedic surgeons, there was no urgent indication for spinal surgery. As the distribution pattern of the metastases, including peritoneal and BM, was not typical for PC, in addition to the PC biopsy, the easily accessible axillary lymph node was biopsied. Core biopsy of the prostate revealed a prostate acinar adenocarcinoma with a Gleason score of 4 + 5, International Society of Urological Pathology grade (ISUP) 5, without evidence of neuroendocrine features. However, several fine needle aspirations of the axillary lymph node showed only necrotic cells, in principle in keeping with metastasis, although this could not be further specified. Due to high specificity for detection of PC, a PSMA PET scan was added, which showed PSMA avidity in all displayed metastases including BM, so that ultimately there was no evidence of suggesting a second tumor entity (**Figure 2**). Under treatment with dexamethasone (initially 12 mg daily), the patient’s CNS symptoms improved rapidly, and after a few days, he was able to walk safely again without ataxia. 4 weeks later, ADT was switched to the GnRH agonist goserelin (10.8 mg every 84 days), and endocrine intensification was commenced with abiraterone (1,000 mg daily in combination with prednisone 5 mg) (15). Meanwhile, dexamethasone has been weaned down without any problems. Repeat staging with MRI brain only 5 weeks after treatment start already showed a substantial remission of the BM with regression of the right frontal (2.4 cm, **Figure 3A**) and left cerebellar lesion (1.2 cm, **Figure 3B**) along with regression of perifocal edema. Established systemic treatment also resulted in a good biochemical response with a serum PSA level decline to 1.1 µg/l. Due to rapid and sufficient response to treatment, no local treatment (radiotherapy, surgery) for BM has been performed so far.

**Figure 4:**
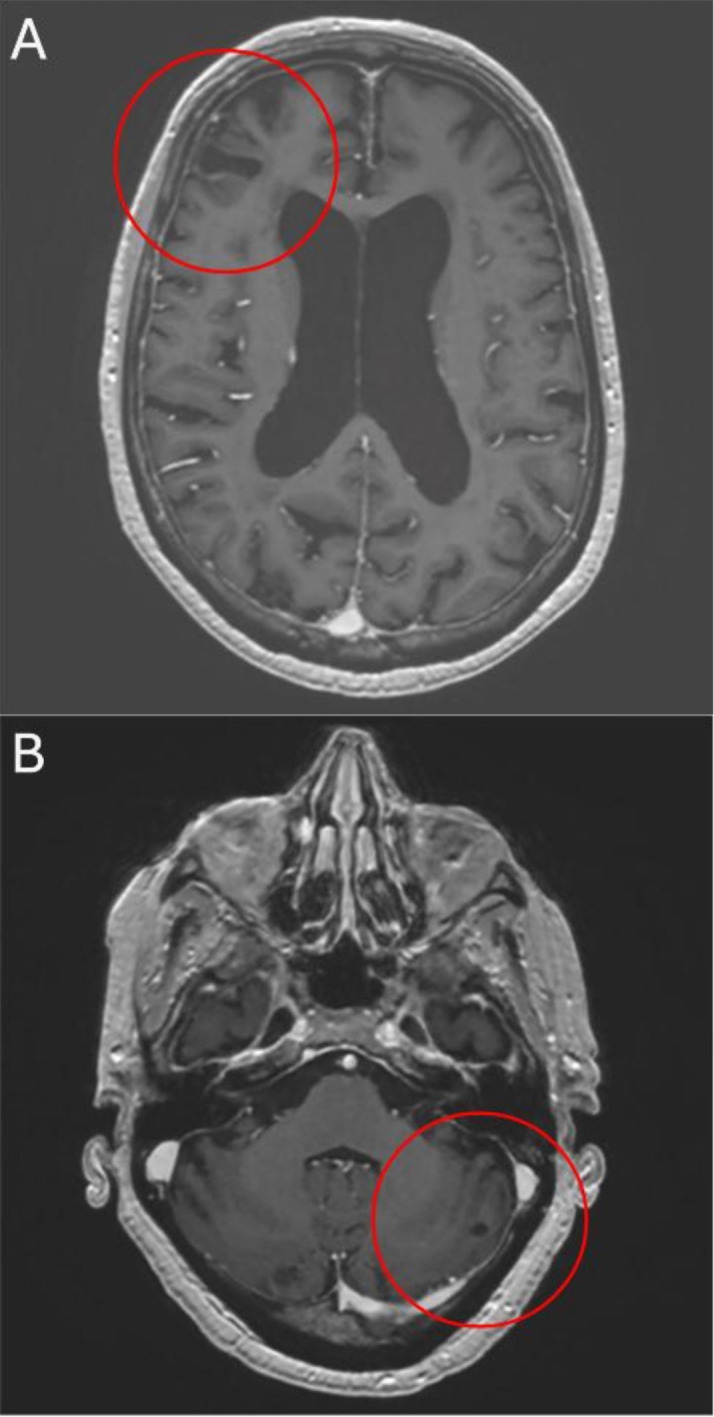
After 11 months of treatment axial T1-weighted MRI shows further reduction of right frontal **(A)** and left cerebellar **(B)** lesions.

The patient’s subjective tolerability of systemic treatment is still unremarkable with no relevant side effects. Eleven months after primary diagnosis and treatment start, follow-up imaging revealed further improvement. CT scans showed another marked size decrease of thoraco-abdominal lymph node metastases, while bone scintigraphy displayed lower metabolic activity of disseminated skeletal metastases over the course. Moreover, an MRI head revealed further decreasing BM with a right frontal lesion measuring 1.2 cm (**Figure 4A**) and a left cerebellar lesion of 0.5 cm (**Figure 4B**), corresponding to only about 30% of the initial BM size. After more than 12 months of abiraterone and ADT, the patient is asymptomatic and has an excellent performance status.

Timeline: Changes of serum PSA level and radiographic response of right frontal CNS metastasis during treatment with GnRH antagonist degarelix and GnRH agonist goserelin plus androgen biosynthesis inhibitor abiraterone/prednisone.

## Discussion

BM is an infrequent site of metastatic PC (12). The estimated incidence in PC patients is less than 2% (16). In particular, first presentation with BM at initial diagnosis is uncommon. According to literature, BM occur at a median range of 6–90 months after initial diagnosis of PC (17). In the vast majority of cases, advanced pre-treated disease with castration resistance is present (6, 7). Most patients only present with single brain metastasis and not several, as in our case (17). Special histological subtypes, usually small cell carcinoma, rather than adenocarcinoma as in our patient are associated with an increased likelihood of BM development (12). Accordingly, even though in absolute numbers most BM from PC are adenocarcinomas, the probability of developing BM in the course of small cell PC is much higher (18). Small-cell and dedifferentiated neuroendcocrine PC are considered aggressive variants and represent a subset of usually castrations-resistant PC, which to a certain extern show an androgen receptor-independent phenotype. Apart from histological features there are other aggressive variant criteria such as low PSA and bulky disease, high LDH, high carcinoembryonic antigen (CEA), visceral metastases, lytic bone metastases and unfavorable genomics (PTEN, TP53, and/or RB1 alterations) (19, 20).

The serum LDH level in our patient was only slightly elevated. In contrast, our patient had a very high PSA at primary diagnosis. Histology revealed acinar adenocarcinoma without neuroendocrine features. The CEA level was not determined. Tumor next-generation sequencing revealed no PTEN, TP53 and/or RB1 alterations. However, a Gleason Score of 9 (ISUP 5) in our patient may reflect the aggressive potential of the PC.

Synchronous visceral metastases are associated with an increased rate of BM as well, as in our patient with presence of peritoneal metastases (17). In this context, it should be mentioned that peritoneal carcinomatosis from prostate cancer is also a rarity. However, limited sensitivity of detecting peritoneal metastases by cross-sectional imaging compared to solid organ metastases must be considered.

As BM are rare in PC, there are no established guidelines for their treatment. Local therapies are common options, but given its rarity only case reports or case series are reported on surgery, stereotactic radiotherapy and whole-brain radiotherapy (9, 21, 22). Of note, death often does not seem to result from involved brain site but rather from progression of additional metastatic disease sites (23). The exclusion of patients with BM from most clinical trials and the lack of model systems hamper progress in BM treatment. While these issues are important, the major obstacle to effective treatment is the BBB, as most oncological systemic treatments cannot pass this barrier (24). Correspondingly, there is almost no uptake of docetaxel in the brain in PC patients during therapy (25) and thus chemotherapy may not have a significant effect on survival of PC patients with BM. Comparably, practically all PARP inhibitors do not pass the BBB, nor does the radionuclide radium 223 (26–29). The newer taxane cabazitaxel appears to penetrate the BBB better in mouse models, but it is still unclear to what extent this leads to intracerebral efficacy of cabazitaxel in BM from PC (30). At least case reports on the efficacy of cabazitaxel in newly diagnosed BM from PC have been published (31). CNS side effects including seizure risk indicate that the androgen receptor pathway inhibitors (ARPIs) enzalutamide and apalutamide achieve at least low intracerebral drug concentration (32, 33). In contrast, the newer ARPI darolutamide shows negligible CNS penetration, so that no CNS side effects are to be expected, making darolutamide generally attractive in this respect for the treatment of PC patients (34, 35).

As stated, due to the lack of CNS penetration (docetaxel, PARP inhibitors, radium223, darolutamid) and other issues with CNS toxicity including seizure activity (enzalutamide, apalutamide) there is no defined role for systemic treatment of BM from PC so far.

Of note, some pharmacokinetic studies have shown that abiraterone passes the BBB and thus could have intracranial activity. However, there is no comprehensive evidence that this results in clinically significant efficacy (36). Abiraterone acts as a selective inhibitor of CYP450 17-alpha hydroxylase, which is a key enzyme in the synthetic pathway of androgens (37). This inhibition leads to the blockade of androgen biosynthesis in the testes, adrenal gland, and prostate as well as in the tumor (38). By lowering serum testosterone levels and therefore the amount of testosterone that passes the BBB, abiraterone may affect PC BM as well.

In conclusion, our patient has had a significant clinical, biochemical, and radiographic response to systemic treatment with abiraterone and ADT. Despite the presence of high-volume high risk metastatic PC, decision was made against a more intensive triple therapy including chemotherapy due to patient`s age (39). Even 12 months after the initial diagnosis of metastatic hormone-sensitive PC with BM, there is a sustained response, which may contradict an initially expected dismal course of disease. Subjective tolerability of the systemic treatment overall is still given. For this reason, local treatment options (surgery, radiotherapy) for BM have not been necessary to date. After broad literature search (PubMed, MEDLINE), the case described is unique. A certain CNS penetration could serve as potential rationale for the efficacy of abiraterone in BM from PC. Since testosterone can overcome the BBB and cause PC cell growth, an abiraterone induced reduction of circulating testosterone levels may explain its efficacy as well. In this context, however, it should be noted that we cannot quantify the effect of abiraterone compared to ADT alone. Nevertheless, in our opinion, abiraterone should be considered in the choice of systemic treatment of BM from PC.

Undoubtedly, there are further limitations to our work. Most obvious, it is difficult to generalize our single case experience of abiraterone efficacy to a broader population of PC patients with BM. Furthermore, although with combination abiraterone and ADT the serum PSA level in our patient has decreased significantly, it was still >0.2 µg/l at 7 months after ADT initiation. There is evidence that PSA levels <0.2 µg/l at 7 months after ADT initiation are prognostic in the metastatic hormone-sensitive setting with longer overall survival, although these data were obtained from a circumscribed patient population without BM treated with docetaxel, so that the transferability in this respect is limited (40).

## Data Availability

The original contributions presented in the study are included in the article/supplementary material. Further inquiries can be directed to the corresponding author.
